# AST-120 Reduces Neuroinflammation Induced by Indoxyl Sulfate in Glial Cells

**DOI:** 10.3390/jcm7100365

**Published:** 2018-10-17

**Authors:** Simona Adesso, Irene Paterniti, Salvatore Cuzzocrea, Masaki Fujioka, Giuseppina Autore, Tim Magnus, Aldo Pinto, Stefania Marzocco

**Affiliations:** 1 Department of Pharmacy, University of Salerno, Via Giovanni Paolo II 132, Fisciano, I-84084 Salerno, Italy; sadesso@unisa.it (S.A.); autore@unisa.it (G.A.); pintoal@unisa.it (A.P.); 2 Department of Chemical, Biological, Pharmaceutical and Environmental Sciences, University of Messina, Viale Ferdinando Stagno D’Alcontres 31, 98166 Messina, Italy; irenepaterniti@gmail.com (I.P.); salvator@unime.it (S.C.); 3 Pharmaceuticals Division, Kureha Corporation, Tokyo 169-8503, Japan; m-fujioka@kureha.co.jp; 4 Department of Neurology, University Medical Center Hamburg-Eppendorf, Martinistraße 52, 20246 Hamburg, Germany; t.magnus@uke.de

**Keywords:** indoxyl sulfate, chronic kidney disease, neuroinflammation, glial cells, AST-120

## Abstract

Chronic kidney disease (CKD) involves multiple organ dysfunction, and the neurological complications that are often present in CKD patients support the idea of a crosstalk between the kidneys and the brain. Evidence suggests a possible role for products accumulating in these patients as uremic toxins in various CKD complications, including neurodegeneration. Indoxyl sulfate (IS), derived from tryptophan metabolism, is well-known as a uremic nephron-vascular toxin, and recent evidence suggests it also has a role in the immune response and in neurodegeneration. Inflammation has been associated with neurodegenerative diseases, as well as with CKD. In this study, we demonstrated that sera of CKD patients induced a significant inflammation in astrocyte cells which was proportional to IS sera concentrations, and that the IS adsorbent, AST-120, reduced this inflammatory response. These results indicated that, among the uremic toxins accumulating in serum of CKD patients, IS significantly contributed to astrocyte inflammation. Moreover, being also chronic inflammation associated with CKD, here we reported that IS further increased inflammation and oxidative stress in primary central nervous system (CNS) cells, via Nuclear Factor-κB (NF-κB) and Aryl hydrocarbon Receptor (AhR) activation, and induced neuron death. This study is a step towards elucidating IS as a potential pharmacological target in CKD patients.

## 1. Introduction

Neurodegenerative diseases are a heterogeneous group of chronic and progressive disorders which are characterized by a gradual loss of anatomically and/or physiologically related neuronal systems. This phenomenon, which mainly affects elderly individuals [1,2], occurs in neurodegenerative diseases, such as Alzheimer’s disease, multiple sclerosis and Parkinson’s disease [3].

Neurodegenerative disorders have also been associated with chronic kidney disease (CKD). CKD patients present several comorbidities, such as immune disorders and neurological complications, which contribute to the morbidity and mortality of these patients [4,5,6]. CKD is frequently associated with cognitive impairments, and it has been estimated that more than 85% of hemodialyzed (HD) patients with terminal CKD have cognitive deficits [7,8,9,10]. The neurological disease in CKD includes symptoms of cognitive slowing, attention deficits, reduced cognitive flexibility, interference susceptibility, verbal and non-verbal memory deficits, language deficits, dementia, and encephalopathy [11].

CKD patients are also characterized by a chronic inflammation state. Multiple factors can contribute to immune dysregulation and inflammatory activation in CKD, some of which could be related to the primary disease rather than to uremia. Other modifiers originate from the genetic background, as well as from the diet, life-style, and environment, representing epigenetic influences. The uremic milieu produces oxidative stress [12] and carbonyl stress [13], and pro-inflammatory and oxidative stress markers were also used to predict response to iron treatment in CKD [14,15]. A condition of chronic inflammation is also believed to affect cognitive function. Specifically, inflammation has been suggested to be an aetiological factor of mild cognitive impairment [16]. A relationship between the levels of C-reactive protein and interleukin-6 (IL-6) and reduced cognitive performance in healthy subjects and in older adults has been reported [17]. In HD patients, it has also been found that abnormal production of pro-inflammatory cytokines is associated with cognitive impairment [18]. Severe or prolonged systemic inflammation can induce harmful changes in cognitive function such as synaptic loss, dendritic alterations, neuronal apoptosis, impaired neurogenesis, memory dysfunction, and altered hypothalamic function, by activating microglia [19].

The uremic syndrome is characterized by the retention of various solutes, called uremic toxins, that are normally excreted by the kidneys and that negatively interact with biological functions [20]. Several toxins are produced from protein breakdown or from the metabolization of amino acids. Studies regarding neurological disease in CKD have focused their attention on the hypothesis that increased levels of these toxins are also responsible for neurological dysfunction [6,21]. Indoxyl sulfate (IS) is a protein-bound uremic toxin, derived from dietary tryptophan, that accumulates in the serum of CKD patients. It is recognized as a nephro-vascular toxin [22] which induces free radicals formation in endothelial cells [23]. In addition, it has been reported that IS enhances the pro-inflammatory response and oxidative stress in macrophages [24]. IS levels are higher in the brain—being transported by OTA3 channels expressed in the blood-brain barrier [25], and plasma of CKD patients (30 µM)—compared to control subjects (4.7 µM) [26]. Therefore, IS is a likely candidate to trigger cerebral dysfunction [27], and more recently it was also reported that IS induces oxidative stress and inflammation in central nervous system (CNS) cells [6].

In this study, we investigated the effect of IS on astrocyte function, among the various toxins present in serum of CKD patients, and the effect of AST-120, an IS adsorbent. Moreover, we also evaluated the impact of IS on CNS cells in inflammatory conditions, which cronically affect CKD patients.

## 2. Experimental Section

### 2.1. Reagents

All compounds and reagents were purchased from the Sigma Chemicals Company (Sigma, Milan, Italy) unless otherwise stated.

### 2.2. In Vitro Studies on Glioma Cell Line (C6)

#### Glioma Cell Line (C6)

The glioma cell line (C6) was obtained from the American Type Culture Collection (ATCC; Manassas, VA, USA). This cell line was grown in Dulbecco’s Modified Eagle Medium (DMEM), 10% fetal bovine serum (FBS), and 2 mM l-glutamine and penicillin/streptomycin (100 units/0.1 mg/mL) at 37 °C in a 5% CO_2_ atmosphere. Cells were passaged at confluence using a solution of 0.025% trypsin and 0.01% Ethylenediaminetetraacetic acid (EDTA). The C6 cell line was originally derived from rat brain tumors induced by *N*-nitrosomethylurea [28]; these cells are widely used as an astrocyte-like cell line [29] because they have astrocytic, oligodendrocytic, and neuronal properties [30].

### 2.3. Studies on the Effect of Sera of CKD Patients on C6 Astrocytes

This is a post-hoc analysis of a preceding study [31,32]. The study was approved by the Ethics Committee of the Campania Nord and conducted in accordance with the current version of the Declaration of Helsinki. We analysed the sera of eighteen subjects: four healthy people (H), eight CKD patients (CKD), and six CKD dialysed patients (HD; Table 1. IS serum levels were measured at baseline and at the end of the treatment with AST-120, which adsorbs IS thereby reducing its serum concentrations. The reduced IS serum concentrations were evaluated by HPLC analysis as previously reported [6,33,34], data not shown. The AST-120 was kindly provided by the Kureha corporation.

#### 2.3.1. C6 Cells Treatment with Sera of CKD Patients

In our experimental model, we used human serum. For these experiments, we referred to previous studies reporting the effect of human serum samples on cell cultures in order to study the effect of human serum deriving from patients on cellular systems [35]. The C6 cells were plated (5.0 × 10^4^ cells/well) and allowed to adhere for 24 h; thereafter, the medium was replaced with fresh medium and the cells were treated with sera of eighteen subjects: from four healthy people, eight CKD patients, and six CKD dialysed patients (sera dilution 1:50 in culture media), for 1 h alone or in combination with Lipopolysaccharide from *Escherichia coli* (LPS; 1 µg/mL) plus Interferon γ (IFN; 100 U/mL) for 24 h. In our experimental model, we used LPS plus IFN in order to induce a proinflammatory response in astrocytes, as previously reported both from our research group and from others [36,37]. In another set of experiments, the serum samples were incubated with 1 g/dL AST-120 for 3 h at 37 °C, as previously reported [38]. Thereafter, AST-120 bound to IS was settled as a pellet, and the supernatants were filtered and administrated to the C6 cells (diluted 1:50 in culture media) for 1 h alone or in the presence of LPS + IFN for a further 24 h. Reactive oxygen species (ROS), nitric oxide (NO), and tumor necrosis factor-α (TNF-α) release were evaluated, as subsequently reported.

#### 2.3.2. ROS Evaluation in C6 Cells

ROS production was evaluated utilizing the probe 2′,7′-dichlorofluorescin-diacetate (H_2_DCF-DA), as previously reported [39]. In the presence of intracellular ROS, H_2_DCF is rapidly oxidized to the highly fluorescent 2′,7′-dichlorofluorescein (DCF). Briefly, C6 cells were plated at the density of 3.0 × 10^5^ cells/well into 24-well plates. The cells were allowed to adhere for 24 h; thereafter, the medium was replaced with fresh medium and the cells were treated with serum samples as previously described. The cells were then collected, washed twice with phosphate buffered saline (PBS), and incubated in PBS containing H_2_DCF-DA (10 µM) at 37 °C. After 15 min, cellular fluorescence was evaluated using fluorescence-activated cell sorting analysis (FACSscan; Becton Dickinson, Milan, Italy) and elaborated with Cell Quest software (FACSscan; Becton Dickinson, Milan, Italy).

#### 2.3.3. NO Determination in C6 Cells

The C6 cells were plated at a density of 3.0 × 10^5^ cells/well into 24-well plates for 24 h. Thereafter, the medium was replaced and cells were treated with serum samples, as previously described. NO generation was measured as nitrite (NO_2_^−^), index of NO released by cells, in the culture medium by the Griess reaction, as previously reported [37]. Briefly, 100 µL of cell culture medium was mixed with 100 µL of Griess reagent—equal volumes of 1% (*w*:*v*) sulphanilamide in 5% (*v*:*v*) phosphoric acid and 0.1% (*w*:*v*) naphtylethylenediamine-hydrogen chloride and incubated at room temperature for 10 min. The absorbance was measured at 550 nm in the microplate reader Titertek (Dasit, Cornaredo, Milan, Italy). The amount of NO_2_^−^ in the samples, as a µM concentration, was calculated using a sodium NO_2_^−^ standard curve.

#### 2.3.4. TNF-α Determination in C6 Cells

TNF-α concentration in the supernatant of C6 cells stimulated with serum samples as previously described, was assessed by an Enzyme-Linked Immuno Sorbent Assay (ELISA). A commercially-available kit for rat TNF-α was used according to the manufacturer’s instructions (e-Biosciences, San Diego, CA, USA), as previously reported [6].

### 2.4. Ex Vivo Studies on Primary Astocytes, Glial Cells

#### 2.4.1. Primary Astrocytes and Glial Cells

Mixed glial cell cultures from the cortex and spinal cord were prepared from postnatal day 1–2 mouse pups (Female C57BL/6J mice; Harlan Laboratories, Udine, Italy). The mice were fed with a standard chow diet and housed under specific pathogen-free conditions at the University of Messina, Department of Chemical, Biological, Pharmaceutical and Environmental Sciences. The protocols of the Italian and European Community Council for Animal Care (DL. 26/2014) were followed for all animal experiments. The cerebral cortices were excised, the meninges, olfactory bulb and thalami removed, and the hemispheres transferred to Petri dishes containing Hanks’ Balanced Salt solution (HBSS) before being cut into four small pieces. The brains were centrifuged for 1 min at 200–300 g. The supernatant was removed and the pellet was incubated with HBSS/10 mM HEPES buffer, 0.5 mg/mL Papain, and 10 μg DNAse solution for 25 min at 37 °C. The extracted cells were centrifuged for 5 min at 200–300 g and the pellet was re-suspended in BME medium (10% FBS and 0.5% penicillin/streptomycin). The cell suspension was filtered through a 70 µm cell strainer to remove debris. The extracted cells were suspended in Basal Medium Eagle (BME) medium (10% FBS and 0.5% penicillin/streptomycin) in 75 cm^3^ flasks. The medium was changed after 48 h and then twice per week [40]. After 20 days, in some flasks, the microglia were dislodged using an orbital shaker (200 rpm for 1 h, 37 °C). Moreover, to remove residual microglia from the remaining cell monolayers, a 60 min exposure (50 mM) to the lysosomotropic agent Leu-Leu-OMe (<5% microglia, flow cytometry using anti-Iba1 as antibody) was used [41].

#### 2.4.2. Primary Astrocytes, Glial Cells Treatment

Primary astrocytes and mixed glial cell cultures were plated and allowed to adhere for 24 h; thereafter, the medium was replaced with fresh medium and cells were treated with IS (60, 30 and 15 µM) for 1 h and then co-exposed with LPS (1 µg/mL) and IFN (100 U/mL) for 24 h in all experiments, except for Nuclear Factor-κB (NF-κB) nuclear translocation and Aryl hydrocarbon Receptor (AhR) activation where IS and LPS + IFN were added to the cells for 1 h.

Regarding the concentration of IS used in this study, we considered both previous studies and referred to the list of uremic toxins provided by the European Uremic Toxin Work group for the experiments, and thus considered the IS concentration range found in the cerebrospinal fluid of CKD patients [42,43,44].

#### 2.4.3. NO, TNF-α and IL-6, ROS Determination in Primary Astrocytes, Glial Cells

Primary astrocytes and mixed glial cell cultures were plated at a density of 1.5 × 10^5^ cells/well into 24-well plates. Thereafter, the medium was replaced, the cells were treated with IS (60, 30 and 15 µM) for 1h, and then then co-exposed to LPS + IFN for a further 24 h. NO generation was measured as NO_2_^−^ by the Griess reaction, as previously indicated.

TNF-α and IL-6 concentrations in the supernatant of cultured primary astrocytes and mixed glial cells were assessed using an Enzyme-Linked Immuno Sorbent Assay (ELISA). Commercially-available kits for murine TNF-α and IL-6 were used according to the manufacturer’s instruction (e-Biosciences, San Diego, CA, USA).

For ROS determination, primary astrocytes and mixed glial cell cultures were plated at a density of 1.5 × 10^5^ cells/well into 24-well plates. After 24 h, the medium was replaced with fresh medium and the cells were treated with IS (60, 30 and 15 µM) and LPS + IFN, as reported. After cellular treatment, ROS production was evaluated as described for C6 cells.

#### 2.4.4. iNOS, COX-2, Nitrotyrosine and HO-1 Detection in Primary Astrocytes, Glial Cells

Inducible nitric oxide synthase (iNOS), cicloxygenase-2 (COX-2), nitrotyrosine formation, heme oxygenase-1 (HO-1), and in primary astrocytes and mixed glial cell cultures, were assessed using cytofluorimetric analysis. Primary astrocytes and mixed glial cell cultures were plated into 96-well plates (3.5 × 10^4^ cells/well) and were allowed to grow for 24 h at 37 °C in a 5% CO_2_ atmosphere before the experiments. Thereafter, the medium was replaced, the cells were treated with IS (60, 30 and 15 µM) for 1 h, and then co-exposed to LPS + IFN for further 24 h. The cells were collected, washed twice with PBS, then incubated in Fixing Solution for 20 min at 4 °C before being incubated in Fix Perm Solution for 30 min at 4 °C. Anti-iNOS antibody (BD Laboratories), anti-COX-2 antibody (BD Laboratories), anti-nitrotyrosine antibody (Millipore), anti-HO-1 antibody (Santa Cruz Biotechnologies) were then added to the astrocytes and mixed glial cells for a further 30 min. The secondary antibody was added in Fix Perm solution and the cells were evaluated using a fluorescence-activated cell sorting (FACSscan; Becton Dickinson, Milan, Italy) and elaborated with Cell Quest software (FACSscan; Becton Dickinson, Milan, Italy), as previously reported [45].

#### 2.4.5. Immunofluorescence Analysis with Confocal Microscopy for Primary Astrocytes and Mixed Glial Cells

For the immunofluorescence assay, primary astrocytes and mixed glial cells (2.0 × 10^5^/well) were seeded on coverslips in 12 well plate and treated for 1 h with IS (30 µM), and then co-exposed to LPS + IFN for 1 h. The cells were then fixed with 4% paraformaldehyde in PBS and permeabilized with 0.1% Triton X-100 in PBS. After blocking with Bovine serum albumin (BSA) and PBS, the cells were incubated with rabbit anti-p65 NF-κB antibody (Santa Cruz Biotechnologies) or with mouse anti-AhR antibody (Abcam), for 1 h at 37 °C. The slides were then washed with PBS three times, and the fluorescein-conjugated secondary antibody was added for 1 h. 4′,6-diamidino-2-phenylindole (DAPI) was used for counterstaining of the nuclei. The coverslips were finally mounted in mounting medium and fluorescence images were taken using the Laser Confocal Microscope (Leica TCS SP5).

### 2.5. Ex Vivo Studies on Primary Cortical and Hippocampal Neuronal Cultures

#### 2.5.1. Primary Neurons

Primary neurons were obtained from dissociated cell cultures of the mouse cortex, and hippocampus neurons were obtained from day 16 C57BL/6J mouse embryos, as previously described [46]. The cortical and hippocampal neurons were plated in 35, 60, or 100-mm diameter polyethylenimine-coated plastic dishes and maintained at 37 °C in Neurobasal medium containing 25 mM of glucose and B-27 supplement (Invitrogen), 2 mM l-glutamine, 0.001% gentamycin sulfate, and 1 mM HEPES (pH 7.2). Approximately 95% of the cells in these cultures were neurons; the remaining cells were astrocytes.

#### 2.5.2. Primary Cortical and Hippocampal Neuronal Cells Treatment

Primary cortical and hippocampal neuronal cultures were plated and cultured for two weeks; thereafter, the medium was replaced and the cells were treated with supernatant from primary astrocytes and mixed glial cultures treated with IS (60, 30 and 15 µM) for 24 h.

#### 2.5.3. Cytotoxicity Assay on Primary Cortical and Hippocampal Neuronal Cultures

The cytotoxicity assay was performed using the Cytotoxicity Detection KitPLUS LDH (Roche) according to the manufacturer’s instructions. This assay is based on the measurement of lactate dehydrogenase (LDH) activity released from the cytosol of damaged cells. Cell cytotoxicity was studied using LDH activity on primary cortical neuronal cultures treated with the supernatant deriving from IS (60, 30 and 15 µM) treated primary astrocytes and mixed glial cells. Three controls were included: background control (assay medium), low control (untreated cells), and high control (maximum LDH release). To determine the experimental absorbance values, the average absorbance values of the triplicate samples and controls were calculated and subtracted from the absorbance values of the background control. The percentage of cytotoxicity was determined using the following equation: Cytotoxicity (%) = (exp. value − low control)/(high control − low control) × 100.

### 2.6. Data Analysis

Data are presented as the standard error of the mean (s.e.m.) showing the combined data of at least three independent experiments each in triplicate. Statistical analysis was performed by an analysis of variance test, and multiple comparisons were made by Bonferroni’s test. A *p*-value lower than 0.05 was considered significant.

## 3. Results

### 3.1. Sera of CDK Patients Increased ROS, Nitrite and TNF-α Production in a IS-Concentration Related Manner

**Figure 1:**
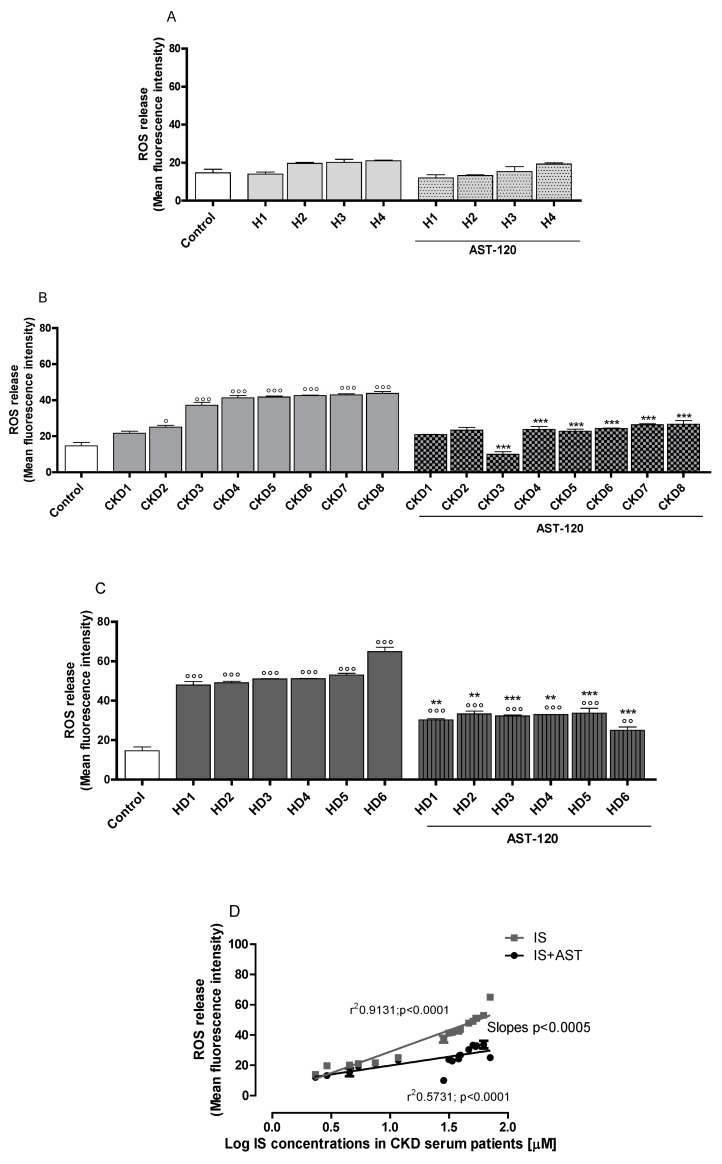

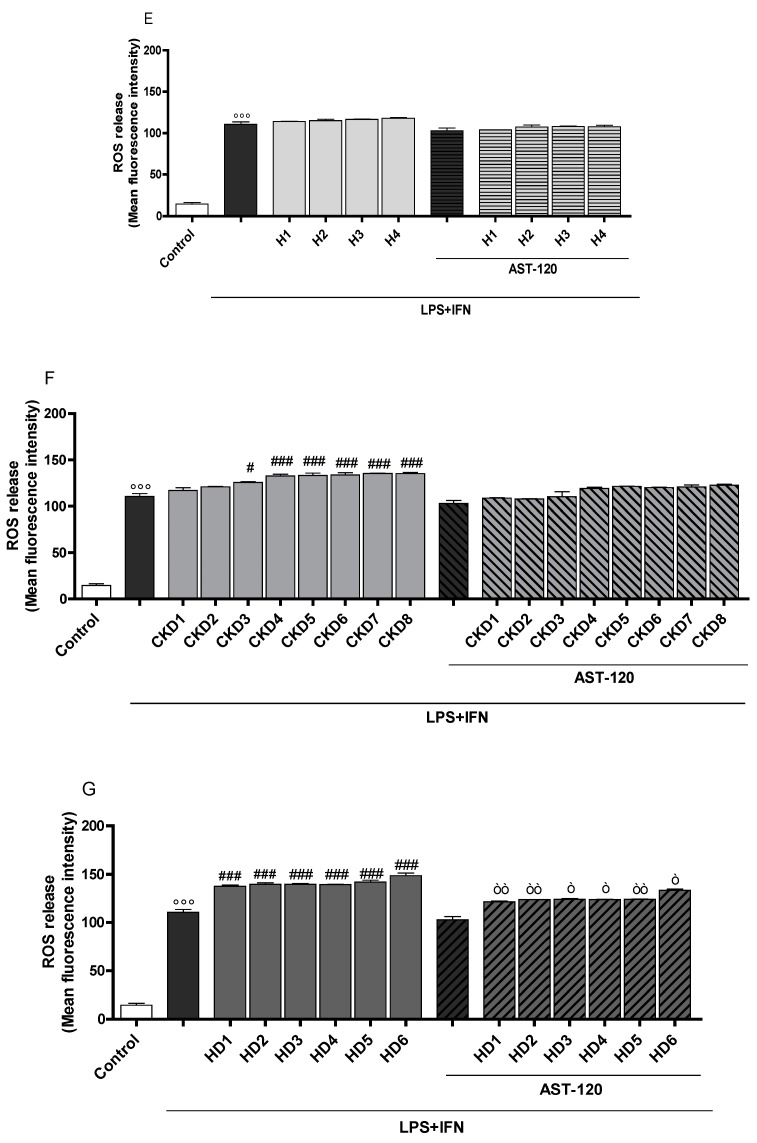
Effect on ROS production in C6 astocytes of sera from eighteen different subjects: four human healthy people (H1–H4; **Panel A**), eight CKD patients (CKD1–CKD8; **Panel B**), and six CKD dialysed patients (HD1–HD6; **Panel C**), also treated with AST-120. In order to highlight the relationship between ROS release and IS serum levels, a linear regression analysis was performed. Linear regression of ROS release by C6 cells treated with sera alone or in the presence of AST-120, indicated F = 52.65, DFn = 1, DFd = 68, *p <* 0.0001; the difference between the slopes are considered extremely significant (**Panel D**). The effect of the eighteen tested sera, also treated with AST-120, on ROS production in C6 astrocytes during inflammatory conditions, induced by LPS (1 µg/mL) + IFN (100 U/mL) is shown (**Panel E**, **F**, and **G**). Comparisons were performed using a one-way analysis of variance and multiple comparisons were made by Bonferroni’s test. °°°, °° and ° denote *p <* 0.001, *p <* 0.05 vs. control. ***, ** denote *p <* 0.001, *p <* 0.01 vs. serum alone. ###,# denote *p <* 0.001, *p <* 0.05 vs. LPS + IFN. òò, ò denote *p <* 0.01, *p <* 0.05 vs. serum + LPS + IFN.

In order to assess the effect of sera of CKD patients on ROS, NO, and TNF-α release, in C6 cells, we treated the cells with sera from eighteen different subjects: four healthy people (H1, IS = 2.32 μM; H2, IS = 2.91 μM; H3, IS = 4,52 μM; H4, IS = 5.38 μM), eight CKD patients (CKD1, IS = 7.48 μM; CKD2, IS = 11.70 μM; CKD3, IS = 28.44 μM; CKD4, IS = 31.46 μM; CKD5, IS = 33.67 μM; CKD6, IS = 38.13 μM; CKD7, IS = 38.73 μM; CKD8, IS = 39.55 μM) and six CKD dialysed patients (HD1, IS = 46.50 μM; HD2, IS = 50.44 μM; HD3, IS = 53.23 μM; HD4, IS = 54.25 μM; HD5, IS = 62.08 μM; HD6, IS = 70.74 μM). Our results indicated that ROS release was not significantly influenced in C6 cells treated with sera of human healthy people (H1–H4; Figure 1A), but resulted increased in the cells treated with sera of CKD patients (CKD1–CKD8; Figure 1B), and mostly in the cells treated with CKD dialysed patients serum (HD1–HD6; Figure 1C), both in normal (Figure 1A–C), and in inflammatory conditions (Figure 1E–G). It is worth noting that it was observed that there is a correlation between IS serum concentration and ROS production in serum-treated astrocytes. In fact, serum-induced ROS release in astrocytes was related to IS content in the sera of CKD patients, as indicated by r^2^ values and the difference between the slopes (Figure 1D). In order to evaluate the specific contribution of IS among all the uremic toxins accumulating in CKD serum, an IS-adsorbent AST-120 was used in some experiments. AST-120 serum treatment significantly reduced ROS release from astrocytes both in normal, and in inflammatory conditions (Figure 1A–C,E–G), thus indicating the significant serum-induced ROS release IS contribution to the observed effects.

**Figure 2:**
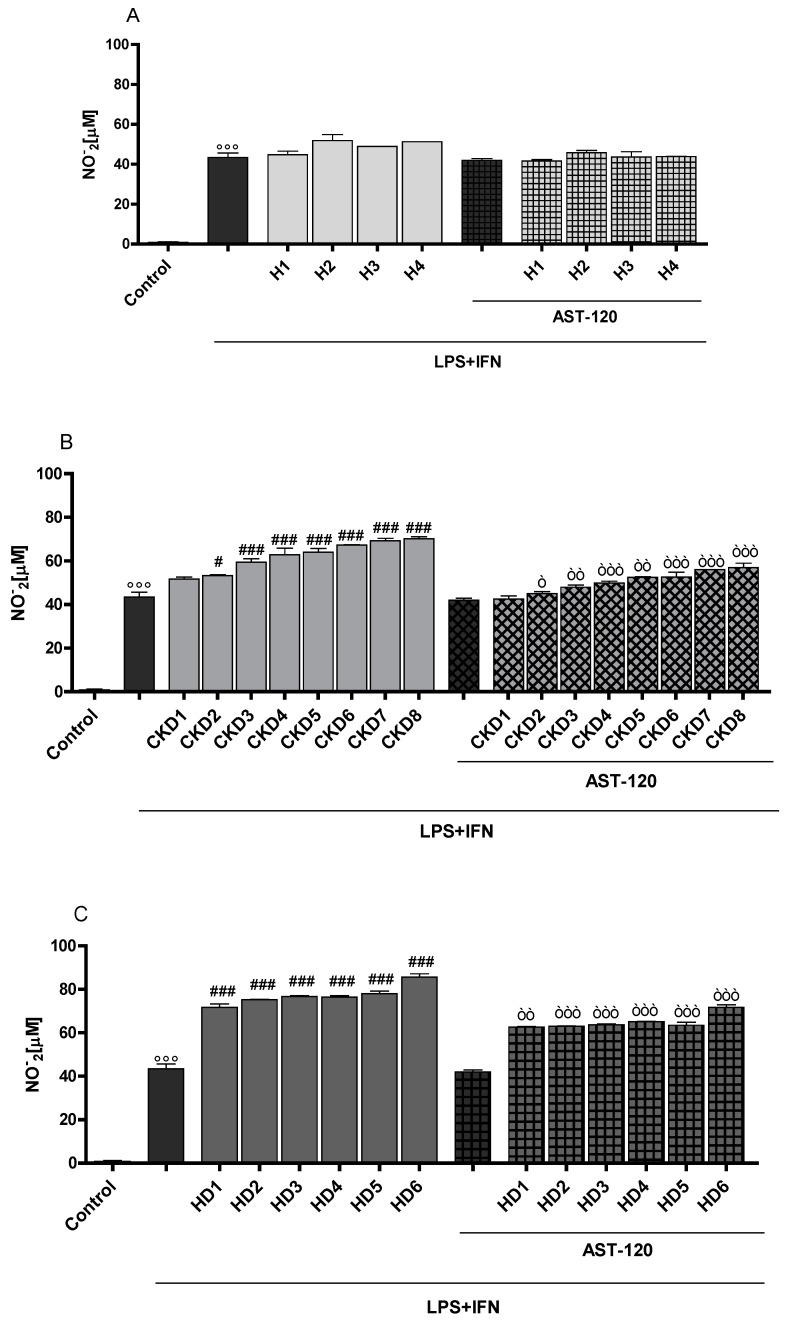

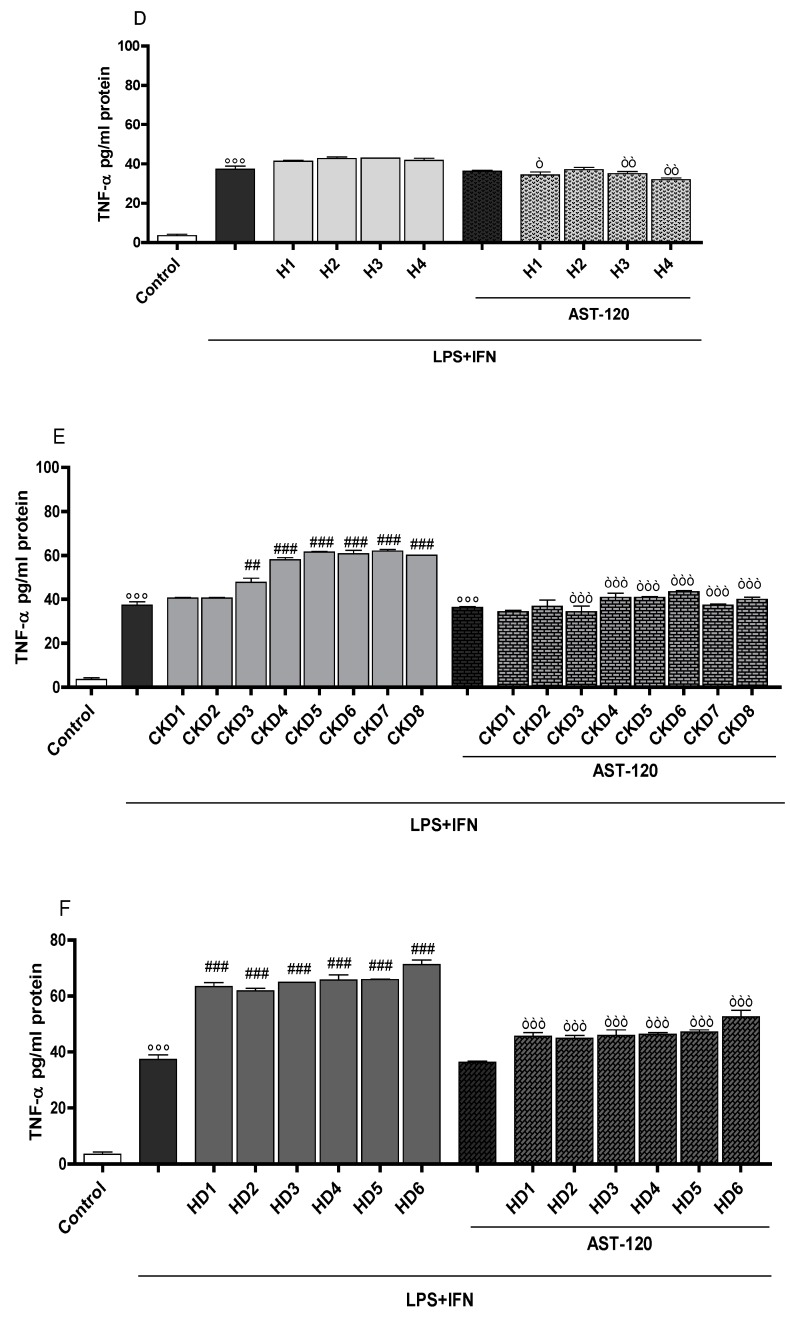
Effect of sera from eighteen different subjects: four healthy people (H1–H4; **Panel A** and **D**), eight CKD patients (CKD1–CKD8; **Panel B** and **E**) and six CKD dialysed patients (HD1–HD6; **Panel C** and **F**), with or without AST-120, on nitrite (**Panel A**–**C**) and on TNF-α (**Panel D**–**F**) production in inflammatory conditions, induced by LPS (1 µg/mL) and IFN (100 U/mL) in C6 cells. Values are expressed as [µM] NO_2_^−^ release, or as pg/mL protein, respectively. Comparisons were performed using a one-way analysis of variance and multiple comparisons were made by Bonferroni’s test. °°° denotes *p <* 0.001 vs. control. ###, ##, # denote *p <* 0.001, *p <* 0.01, *p <* 0.05 vs. LPS + IFN. Òòò, òò, ò denote *p <* 0.001, *p <* 0.01, *p <* 0.05 vs. serum + LPS + IFN.

NO and TNF-α release were not significantly influenced in C6 cells treated with sera from human healthy people (H1-H4; Figure 2A,D), but resulted increased in the cells treated with sera from CKD patients (CKD1–CKD8; Figure 2B,E) and of CKD dialysed patients (HD1–HD6; Figure 2C,F) in inflammatory conditions. AST-120 serum treatment significantly reduced NO and TNF-α release from astrocytes (Figure 2A–F, respectively), thus highlighting that IS contributes, among the various uremic toxins present in serum of CKD patients, to NO and TNF-α release from astrocytes during inflammation.

### 3.2. Indoxyl Sulphate Enhanced NO Release, iNOS and COX-2 Expression in Inflammatory Conditions in CNS Cells

**Figure 3:**
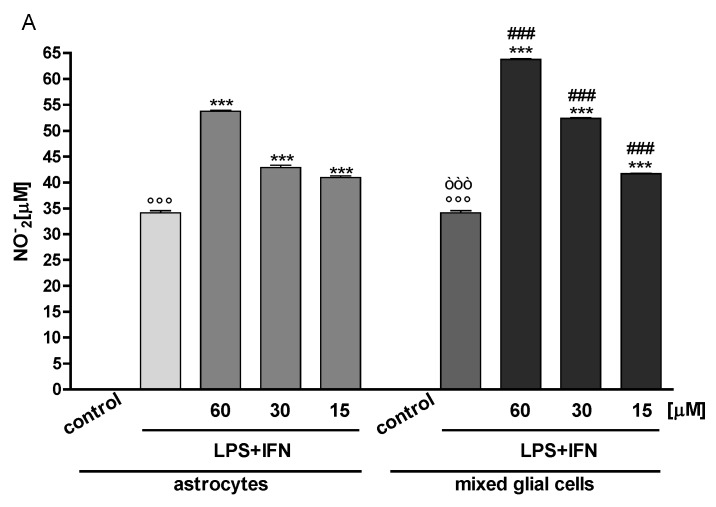

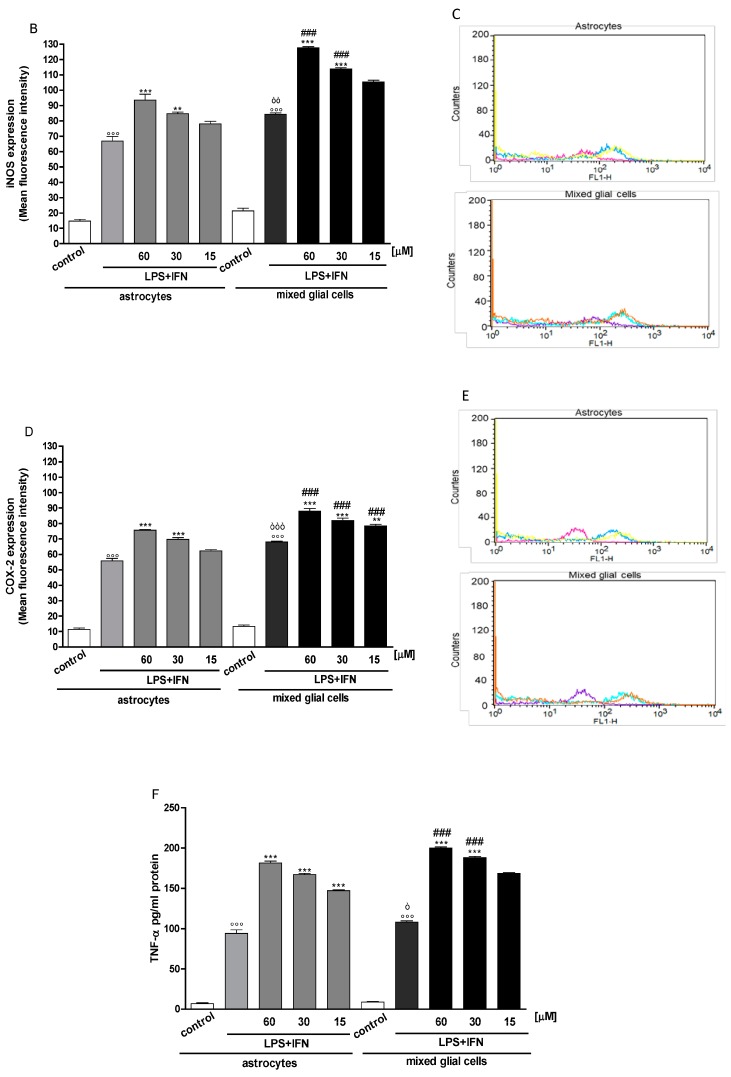

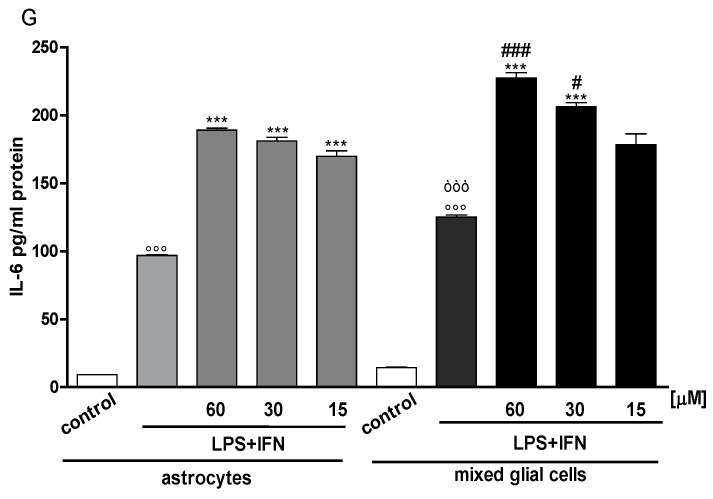
Effect of IS (60, 30 and 15 μM) in inflammatory conditions, induced by LPS (1 µg/mL) + IFN (100 U/mL), on NO relase (**Panel A**), evaluated as NO_2_ [µM]. Effect of IS (15–60 μM) in presence of LPS (1 µg/mL) + IFN (100 U/mL) on iNOS (**Panel B**), and COX-2 (**Panel D**) expression in primary astrocytes and mixed glial cells. Cellular fluorescence was evaluated using fluorescence-activated cell sorting analysis (FACSscan; Becton Dickinson), and elaborated with Cell Quest software. **Panel C** shows the representative fluorescence images for iNOS expression (for primary astrocytes the pink line represent the cellular control, the blue line represent LPS + IFN, the yellow line represent IS 30 µM + LPS + IFN; for mixed glial cells the violet line represent the cellular control, the light blue line represent LPS + IFN, the orange line represent IS 30 µM + LPS + IFN). **Panel E** shows the representative fluorescence images for COX-2 expression (for primary astrocytes the pink line represent the cellular control, the blue line represent LPS + IFN, the yellow line represent IS 30 µM with LPS + IFN; for mixed glial cells the violet line represent the cellular control, the light blue line represent LPS + IFN, the orange line represent IS 30 µM with LPS + IFN. Effect of IS (15, 30, and 60 μM) in inflammatory conditions, induced by LPS (1 µg/mL) and IFN (100 U/mL) on TNF-α (**Panel F**) and on IL-6 (**Panel G**) release by astrocytes and mixed glial cells. Cyokine release was assessed by ELISA assay. Values are expressed as NO_2_^-^ release, or mean fluorescence intensity or as pg/mL protein for cytokines. Comparisons were performed using a one-way analysis of variance and multiple comparisons were made by Bonferroni’s post test. °°° denotes *p <* 0.001 vs. control. ***, ** denote *p <* 0.001 and *p <* 0.01 vs. LPS + IFN. òòò, òò, ò denote *p <* 0.001, *p <* 0.01 and *p <* 005 vs. astrocytes treated with LPS + IFN. ^###^, ^#^ denote *p <* 0.001 and *p <* 0.05 vs. astrocytes treated with IS andLPS + IFN.

In order to evaluate the effect of IS on some pro-inflammatory mediators release also in primary CNS cells, we used mouse primary astrocytes and mouse primary mixed cells for the experiments. The first pro-inflammatory mediator that we evaluated was NO, which plays a key role in the pathogenesis of neuroinflammation. When IS (60, 30 and 15 µM) was added to cells 1 h before and together with LPS + IFN for a further 24 h, a significant increase of NO production in the cell medium was observed in astrocytes, but mostly in mixed glial cells (Figure 3A). NO is synthesized by iNOS, and together with the COX-2 pathway, can modulate the inflammatory response in CNS. Under the same experimental conditions, we observed a significant induction in iNOS and COX-2 expression in primary astrocytes and mostly in mixed glial cell cultures treated with IS + LPS + IFN, compared to LPS + IFN alone (Figure 3B–E).

### 3.3. Indoxyl Sulphate Enhanced TNF-α, IL-6 Production in Inflammatory Conditions in CNS Cells

The inflammatory process is also characterized by an over-production of some cytokines, such as TNF-α and IL-6, which play a significant role in CNS immune and inflammatory activities. During the neuroinflammatory process, microglia, and astrocytes became activated, and they also increase the release of these two cytokines, thus inducing neuronal death and contributing to the pathogenesis of neurodegenerative diseases. In our experimental conditions, LPS + IFN produced a significant induction in TNF-α and IL-6 release both in primary astrocytes and in mixed glial cell cultures. IS treatment (60, 30 and 15 µM) further increased cytokine release in inflammatory conditions at all tested concentrations (Figure 3F,G). The effect of IS on the pro-inflammatory cytokines release in this case was more evident in primary mixed glial cell cultures compared to primary astrocytes.

### 3.4. Indoxyl Sulphate Enhanced Nitrotyrosine Formation in Inflammatory Conditions in CNS Cells

NO can react with superoxide anions, producing the toxic oxidizing agent peroxynitrite. Peroxynitrite induces nitration of tyrosine residues, leading to the formation of nitrotyrosine and to changes in protein structure and function. Nitrotyrosine is identified as an indicator or marker of cell damage, nitrosative stress, and inflammation.

**Figure 4:**
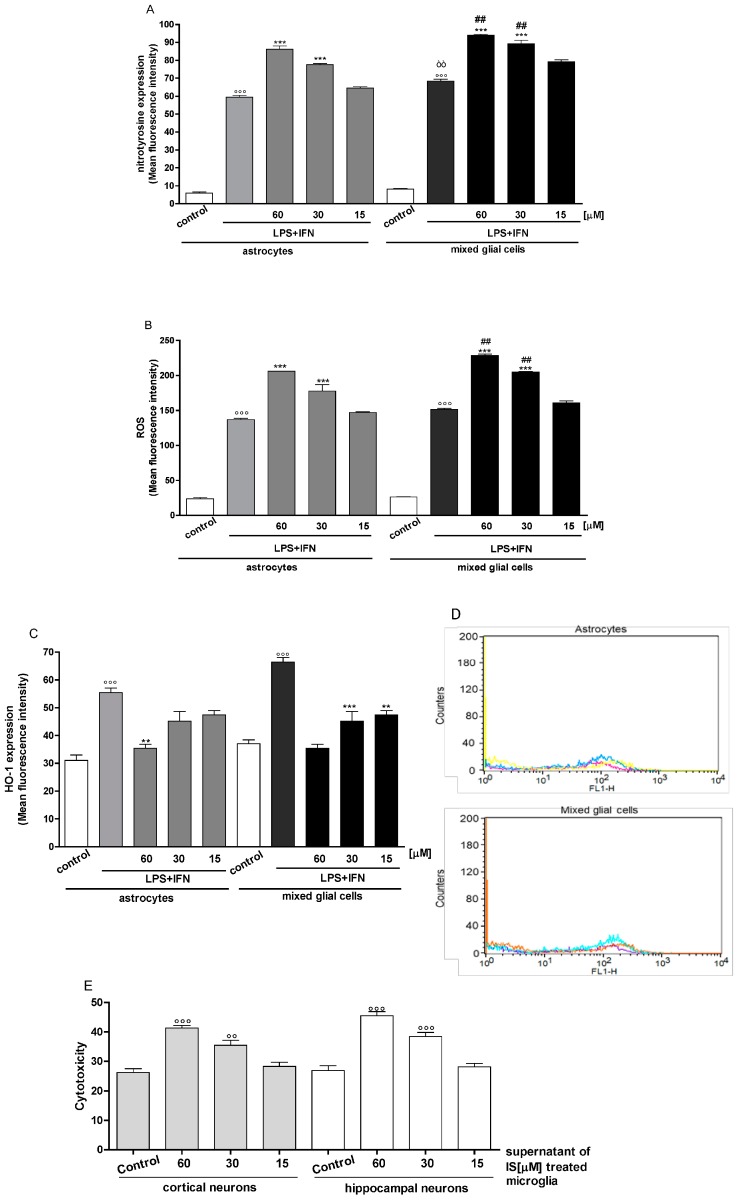
Effect of IS (60, 30 and 15 μM) on primary CNS cells treated with LPS (1 µg/mL) + IFN (100 U/mL) on nitrotyrosine formation (**Panel A**), and on ROS release (**Panel B**), evaluated by means of the probe 2’,7’ dichlorofluorescein-diacetate (H_2_DCF-DA) and on HO-1 expression (**Panel C**) in prmary astrocytes and mixed glial cells. Cellular fluorescence was evaluated using fluorescence-activated cell sorting analysis (FACSscan; Becton Dickinson) and elaborated with Cell Quest software. **Panel D** shows the representative fluorescence images for HO-1 expression (for primary astrocytes the pink line represent the cellular control, the blue line represent LPS + IFN, the yellow line represent IS 30 µM in presence of LPS + IFN; for mixed glial cells the violet line represent the cellular control, the light blue line represent LPS + IFN, the orange line represent IS 30 µM + LPS + IFN). Panel E shows the effect of supernatant from IS-treated microglia on cortical and hippocampal neuronal cell viability. Values are expressed as mean fluorescence intensity or as cell citotoxicity. Comparisons were performed using a one-way analysis of variance and multiple comparisons were made by Bonferroni’s post test. °°°, °° denote *p <* 0.001 and *p <* 0.01 vs. control. ***, ** denote *p <* 0.001 and *p <* 0.01 vs. LPS + IFN. òò denotes *p <* 0.01 vs. astrocytes treated with LPS + IFN. ^##^ denotes *p <* 0.01 vs. astrocytes treated with IS + LPS + IFN.

In order to assess whether IS influenced nitrotyrosine formation in primary CNS cells, we treated astrocytes and mixed glial cell cultures with IS (60, 30 and 15 µM) for 1 h and then co-exposed with LPS + IFN for a further 24 h. In these experimental conditions, nitrotyrosine formation was increased in IS-treated primary CNS cells in inflammatory conditions (Figure 4A). In primary mixed glial cell cultures, nitrotyrosine formation increase was more prominent compared to the primary astrocytes alone.

### 3.5. Indoxyl Sulphate Enhanced ROS Release and HO-1 Expression in Inflammatory Conditions in CNS Cells

Oxidative stress has been associated with myriad pathological conditions, including brain injury and inflammation. Oxidative damage is induced by an increase of ROS levels. In order to investigate the effect of IS on oxidative stress in primary astrocytes and mixed glial cell cultures in inflammatory conditions, we evaluated intracellular ROS production. Our results showed that IS, further increased ROS release during inflammation, compared to LPS + IFN treated cells alone (Figure 4B). In particular, this effect was more evident in primary mixed glial cell cultures compared to primary astrocytes alone, especially at the two highest IS concentrations.

During the increase of pro-oxidant species, it is also important to evaluate the anti-oxidant response associated with the cellular capacity to balance ROS over-production in order to avoid oxidative stress conditions. HO-1 is an enzyme dealing with oxygen radicals which induces an anti-oxidant response. We assessed its expression profile in the presence of IS (60, 30 and 15 µM) during inflammatory conditions. After 24 h, we observed that IS in presence of LPS + IFN induced a decrease in HO-1 expression, compared to LPS + IFN alone (Figure 4C,D). In primary mixed glial cells, the response was much more prominent compared with the primary astrocytes, indicating mostly that the microglial cells were significantly impaired in their antioxidant systems by IS.

### 3.6. Indoxyl Sulphate Supernatant from Glial Cells and Increased Neuronal Death

Activated microglia and astrocytes participate in the inflammatory response increasing the production of inflammatory cytokines, enhancing ROS generation, and therefore resulting in neuronal injury and neuronal loss in the CNS [47,48]. In order to investigate the effect of the supernatants derived from IS-treated microglia on neuronal death, we used primary cortical and hippocampal neuron cultures. Primary neurons were treated with the cells’ supernatant derived from IS-treated microglia. As showed in Figure 4E, in this experimental condition, we observed an increase in cellular mortality in both cortical and hippocampal neurons.

### 3.7. Indoxyl Sulphate Enhanced NF-κB p65 Nuclear Translocation and AhR Activation, Induced by LPS + IFN in CNS Cultures

Upstream of the production of the mediators involved in the inflammatory response and in the oxidative stress which we have assessed so far (NO, iNOS, COX-2, TNF-α, and IL-6), NF-κB nuclear translocation plays a primary role.

**Figure 5:**
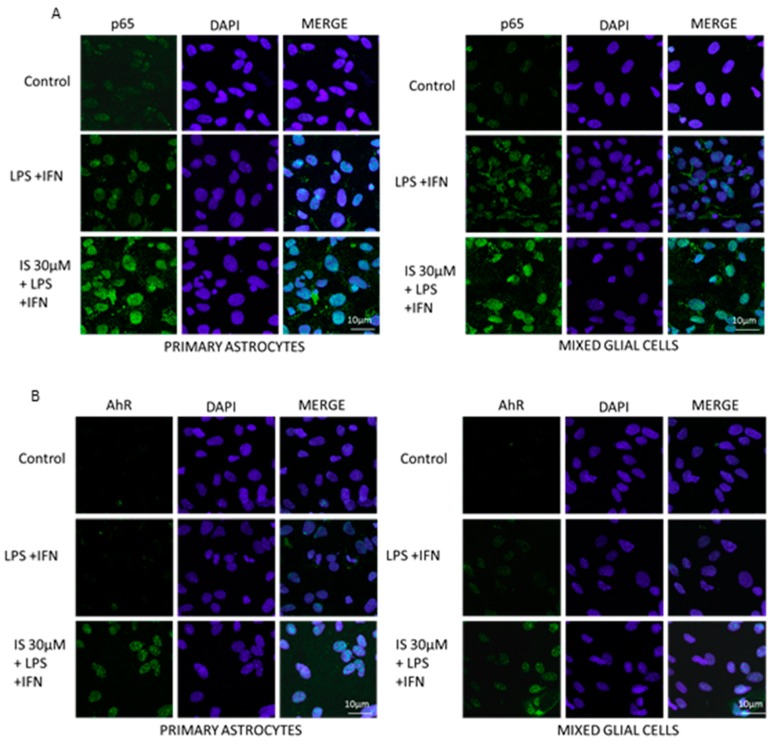
Effect of IS (30 μM) on p65 (**Panel A**) and on AhR (**Panel B**) nuclear translocation in astrocytes and mixed glial cells in inflammatory conditions, induced by LPS (1 µg/mL) + IFN (100 U/mL). Nuclear translocation of p65 and AhR was detected using immunofluorescence confocal microscopy. The scale bar was 10 µm. Blue fluorescence indicated the localization of the nucleus (DAPI) and green indicated the localization of p65 or AhR.

After p65 NF-κB phosphorylation, the free NF-κB dimers translocate into the nucleus and bind to specific sequences to regulate downstream genes expression [49]. Thus, we labelled the p65 subunit with a green fluorescence probe to track the influence of IS (30 µM) in presence of LPS + IFN, on NF-κB translocation. As shown in Figure 5A, nuclear p65 was increased by IS treatment when compared to LPS + IFN alone-treated cells.

It has been reported that IS is an agonist of the AhR receptor [50]. AhR is a nuclear transcription factor involved in multiple processes, such as xenobiotics metabolism and the inflammatory process. Therefore, we investigated AhR through green fluorescent labelling under normal conditions, in the presence of IS (30 µM) and LPS + IFN. After 1 h the nuclear presence of AhR was increased following IS treatment in inflammatory conditions in astrocytes and mixed glial cell cultures (Figure 5B).

## 4. Discussion

CKD patients are characterized by a state of systemic chronic inflammation and oxidative stress. The inflammatory response in CKD patients is tightly linked with the progression of the disease, and this correlates with the level of renal function and also with IS levels. Furthermore, the antioxidant systems are severely impaired in CKD patients and worsen progressively with the degree of renal failure [51]. Thus, the pharmacological manipulation of inflammation, and the resulting oxidative stress control, are important in the management of the uremic syndrome.

CKD has also been associated with neurodegenerative disorders. As the most abundant glial cells in the CNS, astrocytes play an important role in the homeostatic control of the extracellular environment. These cells provide structural, trophic, and metabolic support to neurons, and modulate synaptic activity. Astrocytes are also involved in multiple brain functions, contributing to neuronal development and actively participating in processes triggered by brain injuries [52,53]. Moreover, they are involved in various forms of dementia [54] and in a wide variety of neurological disorders, including neurodegeneration [55].

Many uremic toxins have been proposed to significantly contribute to the neurological disorders observed in CKD. Among these uremic toxins, several guanidino compounds, such as creatine, phosphocreatine, guanidinoacetic acid, creatinine, methylguanidine (MG), guanidinosuccinic acid, g-guanidinobutyric acid, and others are present in the mammalian brain at individual concentrations in the nanomolar to millimolar range [56,57]. These compounds are known to be endogenous convulsants [58], and to induce paroxysmal discharges or tonic-clonic convulsions when administered into the cerebral ventricle or cortex of rodent brains [58]. Our research group reported that MG could contribute to neurodegeneration associated with uremia by enhancing the pro-apoptotic effect of hydrogen peroxide in glial cells [39]. Moreover, MG could also be related to polyneuropathy in uremic dogs [59]. Among the uremic toxins, uric acid also contributed to several CNS diseases, such as bipolar disorders and schizophrenia, and more recently has been also reported to induce neuroinflammation at the hippocampal level [60]. Thus, considering the potential involvement of different uremic toxins in CNS disease, we studied the effect of sera of CKD patients on astrocytes.

In our study, we focused on specific mediators involved in the inflammatory process, which are increased by IS in astrocyte cells such as ROS release, NO, and TNF-α production. The progression of CKD to advanced stages is associated with a significant increase of ROS generation. ROS are produced by various enzymatic reactions and chemical processes, which are essential for many physiological functions. At high concentrations, ROS are deleterious to cells, contributing to the progression of inflammation and oxidative stress. NO is released in response to several stimuli such as proinflammatory agents (cytokines, cell adhesion molecules and growth factors) in CKD patients. The interaction of NO with ROS causes the production of several reactive nitrogen species that potentiate cellular damage [61]. Pro-inflammatory genes and protein expression are up-regulated in CKD patients, with significant increases in the expression of proinflammatory cytokines, such as TNF-α, which further contributes to neuroinflammation. Our data indicated that sera of CKD patients significantly, and in an IS-concentration-related manner, increased the pro-inflammatory response (evaluated by NO and TNF-α release) and the pro-oxidant response (evaluated by ROS release) in astrocytes compared to sera from healthy people. In order to evaluate the specific contribution of IS among all the uremic toxins accumulating in CKD serum patients, we also treated the sera with an IS adsorbent, AST-120. AST-120 is an oral intestinal spherical carbon adsorbent consisting of porous carbon particles that are 0.2–0.4 mm in diameter, and are common organic solvents that are insoluble in water. It is an orally-administered intestinal sorbent that adsorbs IS, thereby reducing the serum and urinary concentrations of IS. AST-120 has a long history of use in Japan and Asia among patients with CKD to prolong the time to initiation of hemodialysis and to improve uremic symptoms. The efficacy of the drug is supported by the results of clinical trials that demonstrate that AST-120 decreases serum IS in a dose-dependent fashion, ameliorates the decline in renal function, and improves at least some symptoms of uremia [62]. However, no data regarding the exposition of astrocytes to sera of CKD patients are available in the scientific literature. Our results indicate that the pro-oxidant and pro-inflammatory response observed treating astrocytes with serum patients was significantly reduced in the presence of AST-120, reaching, in some cases, pro-inflammatory and pro-oxidant mediator’ levels comparable with the sera obtained from healthy people. These results indicate for the first time (among the toxins present in serum from CKD patients) the IS contribution to inflammatory response and oxidative stress in astrocytes. Thus, IS seems to be a uremic toxin of primary importance in inducing inflammation in CKD. Thus, considering that a systemic chronic inflammatory condition is a common feature in CKD patients, to better understand the possible contribution of IS in these conditions, we evaluated the effect of IS on pro-inflammatory mediators primary CNS cells during inflammatory conditions.

In the CNS, the iNOS/NO system forms part of the pathogenesis of neuroinflammatory diseases. NO levels are greatly increased in the CNS during inflammation in both patients and animal models [63]. NO is generated catalytically in tissues by three isoforms of nitric oxide synthase (NOS). The constitutive form, present in neurons (nNOS) and endothelial cells (eNOS), is a calcium-dependent enzyme [64,65]. The inducible form (iNOS), expressed in various cell types including microglia in order to respond to a wide variety of stimuli, is regulated mainly at the transcriptional level, and does not require calcium for its activity [66]. The regulation of iNOS by inflammatory cytokines or LPS has received considerable attention [67]. Our results on primary astrocytes and mixed glial cultures indicated that IS further increased NO production and iNOS expression in inflammatory condition, thus contributing to increased neuroinflammation in CKD. An interaction between iNOS and the COX pathway represents an important mechanism for inflammatory response modulation. COX-2 is expressed in several cell types in response to growth factors, cytokines, and pro-inflammatory mediators. COX-2 over-expression has been associated with neurotoxicity in acute conditions, such as hypoxia/ischemia and seizures, as well as in CNS inflammatory chronic diseases [68]. In our experimental system, we observed that IS increased COX-2 expression compared to LPS + IFN treated primary astrocytes and mixed glial cultures, thereby further enhancing the inflammatory conditions in the CNS. Cytokines production is another feature in neuroinflammation, and TNF-α is a proinflammatory cytokine that has both homeostatic and pathophysiological roles in the CNS [69]. In pathological conditions, microglia release large amounts of TNF-α and scientific evidence has identified TNF-α involvement in the pathogenesis of neurodegenerative disease [69]. Additionally, IL-6 played a role in the neuroinflammatory and functional deficits after injury. Our results show that IS increased TNF-α and IL-6 levels in comparison to LPS + IFN treated primary astrocytes and mixed glial cultures.

Oxidative stress also plays a pivotal role both during neurodegeneration and during neuroinflammation. An increase in oxidative stress and a decrease in the antioxidant capacity of the brain are key factors involved in the pathogenesis of neurodegenerative diseases [70]. Under pathological conditions, increasing ROS production can regulate the expression of diverse inflammatory mediators during brain injury [3]. We found that IS induced a significant and concentration-related ROS release from cultured primary astrocytes and mixed glial cells in inflammatory conditions. These results were also in accordance with previous studies reporting that IS induces oxidative stress by interfering both with pro- and anti-oxidant factors in endothelial cells [23,71], vascular smooth muscle cells [72], kidney cells [73], macrophages [24], and glial cells [6]. In addition, we reported that IS increased nitrotyrosine formation and reduced HO-1, thus impairing protein function and further contributing to decreased cellular antioxidant defences. The observed effects were greater in mixed glial cells compared to primary astrocytes alone, thus highlighting the potential higher neurotoxic IS effect on all glial cell populations. This, in turn, can lead to neuronal apoptosis, one of the most crucial events in neuronal loss occurring in neurological diseases [74,75,76,77]. In fact, in this study, we also observed that the supernatant from IS-treated microglia increased neuronal cell death both in cortical and hippocampal neurons, thereby contributing to neuronal loss and neurodegeneration.

In order to study the mechanisms involved in IS-increased neuroinflammation, we evaluated the activations of NF-κB and AhR. The nuclear factor NF-κB pathway has long been considered a prototypical proinflammatory signalling pathway. NF-κB is an inducible transcription factor, which is also present in neurons and glia. NF-κB can regulate the transcription of genes such as chemokines, cytokines (e.g., TNF-α), proinflammatory enzymes (e.g., iNOS and COX-2), adhesion molecules, inflammasome [78], and other factors in order to modulate neuronal survival [79]. In a previous study, we reported that its activation influenced IS-induced ROS [6]. Pro-inflammatory stimuli, such as LPS and IFN, are known to activate NF-κB, and our data indicated that IS enhanced the phosphorylation and nuclear translocation of the p65 NF-κB subunit, thus improving its activity in primary astrocytes and mixed glial cells during inflammation. These results are in agreement with studies reporting the effect of IS in activating the NF-κB pathway in other cells [80,81]. Furthermore, IS has been reported to be a potent AhR ligand [82]. AhR is expressed in the brain, including the cerebral cortex, hippocampus, and cerebellum [83]. AhR has been implicated in sensorimotor and cognitive dysfunctions caused by oxidative stress or excitotoxicity in neurons [84,85]. Our results showed that IS activated AhR in primary astrocytes and mixed glial cells, and promoted further inflammation by its activation. Our investigation suffers from a two main limitations: (1) the small number of patients, and (2) the study of the correlation between the stage of CKD and the observed response.

## 5. Conclusions

The accumulation of uremic toxins and CKD-associated chronic inflammation are genuine problems in CKD patients. Here, we report that, among the various serum uremic toxins, IS significantly affected astrocytes function, inducing oxidative stress. Moreover, in primary CNS cells in neuroinflammatory conditions, IS further increases the pro-inflammatory response, induces oxidative stress, and factors from astrocytes and microglia capable of reducing neuronal viability, thus contributing to neurodegeneration. In conclusion, we can hypothesize about a significant role for IS in CKD-associated cognitive dysfunction, thus highlighting IS as a potential pharmacological target. IS control (e.g., with specific adsorbent, as AST-120, or with a controlled diet) during CKD could have a significant impact on the treatment of CKD-associated neurological complications.

## Figures and Tables

**Table 1:** Anthropometric data and ematochemical values of the subjects participating in the study.

	Healthy Subjects (H)	CKD Patients (CKD)	CKD Dialysed Patients (HD)
Number	4	8	6
Males	2	5	3
Age, years	51.0 ± 5.8	58.9 ± 12.4	66.4 ± 5.5
Body Weight, kg	77.6 ± 7.9	75.2 ± 12.2	73.5 ± 16.3
Creatinine, mg/dL	0.9 ± 0.2	2.1 ± 0.5	9.8 ± 2.1
Creatinine Clearance	115.0 ± 11.0	37.0 ± 9.0	Not Applicable
Sodium, mmol/L	141.0 ± 4.0	139.0 ± 3.0	142.0 ± 6.0
Kalium, mmol/L	4.5 ± 0.7	4.8 ± 0.6	4.9 ± 0.9
Haemoglobin, g/dL	13.6 ± 0.4	11.2 ± 0.5	10.9 ± 0.6
Albumin, g/dL	4.2 ± 0.1	3.6 ± 0.23	3.5 ± 0.3
